# Antifungal, anti-biofilm, and anti-hyphal properties of *N*-substituted phthalimide derivatives against *Candida* species

**DOI:** 10.3389/fcimb.2024.1414618

**Published:** 2024-06-05

**Authors:** Shamshe Shaik, Jin-Hyung Lee, Yong-Guy Kim, Jintae Lee

**Affiliations:** School of Chemical Engineering, Yeungnam University, Gyeongsan, Republic of Korea

**Keywords:** anti-fungal, biofilm, *Candida*, phthalimide, hyphae, polymicrobial

## Abstract

*Candida* species comprise a ubiquitous pathogenic fungal genus responsible for causing candidiasis. They are one of the primary causatives of several mucosal and systemic infections in humans and can survive in various environments. In this study, we investigated the antifungal, anti-biofilm, and anti-hyphal effects of six *N*-substituted phthalimides against three *Candida* species. Of the derivatives, *N*-butylphthalimide (NBP) was the most potent, with a minimum inhibitory concentration (MIC) of 100 µg/ml and which dose-dependently inhibited biofilm at sub-inhibitory concentrations (10–50 µg/ml) in both the fluconazole-resistant and fluconazole-sensitive *Candida albicans* and *Candida parapsilosis*. NBP also effectively inhibited biofilm formation in other pathogens including uropathogenic *Escherichia coli*, *Staphylococcus epidermidis*, *Staphylococcus aureus*, and *Vibrio parahaemolyticus*, along with the polymicrobial biofilms of *S. epidermidis* and *C. albicans*. NBP markedly inhibited the hyphal formation and cell aggregation of *C. albicans* and altered its colony morphology in a dose-dependent manner. Gene expression analysis showed that NBP significantly downregulated the expression of important hyphal- and biofilm-associated genes, i.e., *ECE1*, *HWP1*, and *UME6*, upon treatment. NBP also exhibited mild toxicity at concentrations ranging from 2 to 20 µg/ml in a nematode model. Therefore, this study suggests that NBP has anti-biofilm and antifungal potential against various *Candida* strains.

## 1 Introduction

*Candida* species are responsible for a large majority of fungal infections in humans. Most of the species in this genus are opportunistic, causing candidiasis. *Candida albicans* is one of the more prominent etiological species that cause candidiasis (Riera et al., 2022) and is responsible for many mucosal and systemic infections in humans (Pappas et al., 2018). Although *C. albicans* exists as a commensal microbe, it is capable of perturbing the host epithelial tissue barrier and evading the host immune responses, thereby causing infections in deep-seated anatomical niches post-asymptomatic colonization of the oral, gastrointestinal, and genital tracts (Lopes and Lionakis, 2022).

*C. albicans* exhibits plasticity in switching between different morphogenic states, which helps with its persistence in various mammalian tissues (Mba et al., 2022). Invasion and damage of the epithelial tissue are possible due to the ability of *C. albicans* to switch from yeast to filamentous (hyphae), which aids invasion (Desai, 2018). Other virulence factors also contribute to candidiasis, such as biofilm formation, systemic signal transduction, hydrolytic enzymes, and toxins (Staniszewska, 2020). Biofilm formation is instrumental in nosocomial-associated infections as it aids in the colonization of both biotic and abiotic surfaces, including medical devices such as stents and catheters (Wijaya et al., 2023).

Biofilm formation commences with the adherence of yeast cells to the surface facilitated by nonspecific factors (e.g., electrostatic forces and cell surface hydrophobicity) or by fungal adhesin and invasion members of the Als (agglutinin-like sequence) and Hwp1 (hyphal wall protein 1) families (Rapala-Kozik et al., 2023). Following the initial adhesion and hyphal proliferation, the accretion of extracellular polymeric substances occurs, followed by the maturation of the biofilm. Some of the non*-*adherent components of the mature biofilm disperse to find a newer site for colonization as yeast cells (Mathé and Van Dijck, 2013). Biofilm formation not only affords *C. albicans* resistance against several clinical antifungals but also acts as a reservoir for recurrent fungal infections (Mathé and Van Dijck, 2013). Inhibition of the hyphal dimorphic switch and biofilm formation, which constitute a prominent component of pathogenesis, can be a viable strategy instead of the fungicidal killing of planktonic cells, as it would mitigate the evolutionary pressure of the development of drug resistance usually associated with traditional antifungals (Kim et al., 2022a). The fluconazole-resistant strain of *C. albicans* was deemed one of the top 18 drug-resistant threats by the Centers for Disease Control and Prevention (CDC; Atlanta, GA, USA), which entails urgent intervention to discover novel antifungal remedies (Kadri, 2020).

Phthalimide analogs comprise one of the important heterocyclic compound groups that possess an array of biological activities, including anticonvulsants, anti-inflammatory, antimycobacterial, and anticancer, among others (Matore et al., 2023). In the past decade, phthalimide has emerged as one of the most important pharmacological scaffolds with two carbonyl groups bound to a secondary amine, imparting its bioactivity (Neelottama Kushwaha, 2016). Some derivatives of phthalimide, such as the ubiquitous Folpet or the agricultural agent Captan, are proven fungicides with broad-spectrum antimicrobial activity against several fungal species (Kluxen et al., 2022). Other synthetic azole and triazine derivatives of phthalimide have also demonstrated antimicrobial activity against several pathogens (Asadi et al., 2023; Yadav et al., 2023). A study demonstrated that the acridine–isoindoline phthalimide derivatives showed an anti-biofilm effect on *Pseudomonas aeruginosa* biofilms (Mane et al., 2019). Moreover, amphiphilic quaternized chitosan with phthalimide functional groups also demonstrated anti-biofilm activity against *Streptococcus mutans* (Vijayakumar et al., 2021; Phuangkaew et al., 2022). Phthalimide derivatives also possess anti-hyphal properties, as in the case of the antifungal phthalimido phenyl urea against fungi such as *Geotrichum candidum* and *Aspergillus fumigatus* (Mohamed and Abd El-Ghany, 2017). Novel thiophthalimide derivatives have also been shown to be potent antimicrobials against *Escherichia coli* and *S. aureus* (Chi et al., 2022). The *N*-aryl and *N*-alkyl derivatives of phthalimides are good anti-mycobacterial agents, and novel bis-phthalimide derivatives have demonstrated significant antibacterial activity against *S. mutans* (Neelottama Kushwaha, 2016). As candidiasis is highly dependent on the formation of hyphae and biofilms, we hypothesized that phthalimide derivatives with anti-biofilm and anti-hyphal activities against other pathogens could be potential antifungal agents against *Candida* species. Therefore, we evaluated the antifungal and antivirulence activities of six phthalimide derivatives against the fluconazole-resistant *C. albicans*. This is the first study to explore the antifungal properties of *N*-substituted phthalimide derivatives against various *Candida* species.

In this study, six phthalimide derivatives against *C. albicans* were investigated for their antifungal and anti-biofilm activities, which showed *N*-butylphthalimide (NBP) as the most potent derivative. The effects of NBP on the planktonic cell growth and viability were assessed. Its anti-biofilm activity, as well as other virulence factors such as hyphal formation and protrusion and cell aggregation associated with candidiasis, was also evaluated. The colony morphology and phenotypic switching on treatment with NBP were confirmed with live cell imaging and scanning electron microscopy (SEM). Molecular insights into the activity of NBP were visualized using quantitative real-time reverse transcription polymerase chain reaction (qRT-PCR). The toxicological fitness of the compound was determined with *Brassica rapa* plant seed germination and using a *C. elegans* nematode model, while its pharmacological fitness was determined using *in silico* ADME (absorption, distribution, metabolism, and excretion) analysis.

## 2 Materials and methods

### 2.1 Strains and chemicals

The *C. albicans* strain DAY185, which is fluconazole-resistant [minimum inhibitory concentration (MIC) > 1,024 µg/ml], was obtained from the Korean Culture Centre for Microorganisms (KCCM), while the fluconazole-sensitive *C. albicans* ATCC 10231 strain was obtained from the American Type Culture Collection (ATCC). *C. albicans* glycerol stock was streaked on potato dextrose agar (PDA) and incubated at 37°C for 48 h. A single colony was used to inoculate 25 ml of potato dextrose broth (PDB) in 250-ml flat-bottomed flasks for 48 h at 37°C. This 48-h culture, hereinafter referred to as the inoculation culture, was used for experiments. *Candida parapsilosis* ATCC 22019 was obtained from the ATCC and grown on PDA. Single colonies were inoculated for the inoculation culture in 2 ml of yeast malt (YM) medium supplemented with 2% glucose for 24 h at 30°C. The uropathogenic *E. coli* (UPEC) O6:H1 strain CFT073 (ATCC 700928) was streaked on Luria–Bertani (LB) agar and incubated in nutrient broth at 37°C for 12 h for overnight culture. *Staphylococcus aureus* (ATCC 6538) and *Staphylococcus epidermidis* (ATCC 14990) were streaked on LB agar and grown in LB broth at 37°C. The *Vibrio parahaemolyticus* strain ATCC 17802 was grown in mineral LB (mLB) broth with 3% (*w*/*v*) NaCl supplementation at 30°C and streaked on mLB agar.

The phthalimide derivatives used in the study were NBP, *N*-methylphthalimide (NMP), *N*-aminophthalimide (NAP), *N*-hydroxymethylphthalimide (NHP), *N*-carbethoxyphthalimide (NCP), and *N*-(2-butynyl)phthalimide (N2BP) (**Supplementary Table S1**). All derivatives were purchased either from TCI Chemicals (Tokyo, Japan) or Sigma-Aldrich (St. Louis, MO, USA) and were dissolved in dimethyl sulfoxide (DMSO) at a concentration not exceeding 0.1% (*v*/*v*). Cell growth was assessed by measuring the turbidity at 600 nm using a spectrophotometer (Multiskan EX microplate reader; Thermo Fisher Scientific, Waltham, MA, USA) post-incubation of the cultures at 37°C for 24 h. The MICs were determined based on the broth dilution method according to the Clinical and Laboratory Standards Institute (CLSI) guidelines (Alastruey-Izquierdo et al., 2015). The *C. albicans* inoculation culture was diluted to ~10^5^ cells/ml and incubated with different concentrations of the six phthalimide derivatives in PDB for 24 h at 37°C in 96-well plates (Rajasekharan et al., 2018; Wang et al., 2021). The MIC was determined as the lowest concentration of the derivative that inhibited microbial growth by both spectrophotometry and colony counting. All experiments were performed with at least two individual cultures in triplicate.

### 2.2 Biofilm inhibition assays

The crystal violet biofilm assay was performed as previously reported (Kim et al., 2022b). The inoculation cultures of both *C. albicans* and *C. parapsilosis* strains were inoculated into fresh PDB and YM medium, respectively, at an initial turbidity of optical density (OD) 0.1 at 600 nm (~10^5^ cells/ml) with or without the phthalimide derivatives at 10, 20, 50, 100, and 200 µg/ml and were cultured for 24 h without agitation at 37°C in 96-well polystyrene plates (Paramasivam Nithyanand et al., 2015). The plates were gently washed with distilled water three times, and 0.1% crystal violet was added and incubated for 20 min before rewashing with distilled water three times. The stained biofilms were solubilized using 95% ethanol, and absorbance was measured using a Multiskan EX microplate reader (Thermo Fisher Scientific, Waltham, MA, USA) at 570 nm (Jeon et al., 2024). The same experiment was performed with the other strains, i.e., UPEC, *S. aureus*, *S. epidermidis*, and *V. parahaemolyticus*, in their respective growth conditions at similar phthalimide treatment concentrations of 10, 20, 50, 100, and 200 µg/ml for 24 h. For the polymicrobial biofilms of *S. epidermidis* and *C. albicans*, *S. epidermidis* inoculated in LB at an initial turbidity of 0.05 at 600 nm was mixed with *C. albicans* inoculated in PDB at an initial turbidity of 0.1 at 600 nm at an equal ratio of 1:1 for a mixed culture. The crystal violet biofilm assay was performed as aforementioned using the mixed culture with or without the phthalimide derivatives at 50, 100, and 200 µg/ml.

### 2.3 Microscopic observation of single and polymicrobial biofilms

Two-dimensional (2D) and 3D representations of the biofilms were constructed as previously described (Kim et al., 2022b). The 96-well plate of the *C. albicans* biofilm was prepared as described in *Section 2.2* with or without NBP at concentrations of 10, 20, 50, 100, and 200 µg/ml at 37°C for 24 h. After incubation for 24 h, the plates were washed gently with distilled water three times, 0.1% crystal violet was added and incubated for 20 min, and washed again gently with distilled water to wash off the previous stain. The plates were visualized for biofilm formation with the iRiS™ Digital Cell Imaging System (Logos Biosystems, Annandale, VA, USA), and color-coded 2D and 3D images of the biofilms were generated using ImageJ software. The experiment was repeated for the polymicrobial biofilm 96-well plate, which was prepared as described in *Section 2.2* with NBP treatment at concentrations of 50, 100, 200, and 300 µg/ml.

SEM was used to investigate biofilm and hyphal formation as previously described (Kim et al., 2022b). Nylon membranes (Whatman, Maidstone, UK) were cut into 0.4 × 0.4-cm pieces and placed in 96-well biofilm plates, prepared as described previously, and then incubated at 37°C for 24 h. The biofilm cells were fixed using a 1:1 mixture of glutaraldehyde (2.5%) and formaldehyde (2%) added at 10% of the total volume of the biofilm culture (*v*/*v*) before incubation for 24 h at 4°C. Post-fixation, the membrane was removed and dehydrated using an ethanol series (50%, 70%, 80%, 90%, 95%, and 100%), followed by critical point drying. The membranes were examined under an S-4800 field-emission scanning electron microscope (FE-SEM; Hitachi, Tokyo, Japan) at a voltage of either 5 or 10 kV and with magnifications ranging from ×500 to ×5,000. Similarly, the SEM for the polymicrobial biofilms of *S. epidermidis* and *C. albicans* was performed in the same conditions as described in *Section 2.2* (Sathiyamoorthi et al., 2024).

### 2.4 Cell survival assay

A cell survival assay was performed by inoculating overnight cultures of UPEC, *S. aureus*, and *V. parahaemolyticus* in their respective media to an initial cell count of ~10^7^ or 10^8^ CFU/ml with or without NBP treatment at concentrations of 0, 200, and 400 µg/ml at 37°C for 24 h. The cell survival of *C. albicans* with NBP treatment was examined at concentrations of 0, 100 and 200 µg/ml at similar conditions. The colony forming units (CFU) on the plates were measured after incubation for 24 h and at 37°C.

### 2.5 Hyphal development and colony morphological assays

The colony morphology of *C. albicans* DAY185 was assessed by spotting 10 µl of the inoculation culture onto solid PDA plates with or without NBP at concentrations of 10, 20, 50, and 100 µg/ml and then incubated in static condition at 37°C for 5 days. The morphology of the colonies was observed under an iRiS Digital Cell Imaging System (Anyang, Republic of Korea).

The cell aggregation and hyphae formation were analyzed as previously described (Zelante et al., 2012). The PDB medium was inoculated at a dilution of 1:50 with the inoculation culture with or without NBP at concentrations of 10, 20, 50, and 100 µg/ml and then incubated in a static condition at 37°C for 24 h. Post-incubation, the cultures were observed for cell aggregation and hyphal formation in bright field using the iRiS Digital Cell Imaging System.

### 2.6 RNA isolation and quantitative real-time PCR

*C. albicans* was inoculated into 50 ml of fresh sterile PDB with or without NBP at 100 µg/ml in 250-ml flat-bottomed flasks using the inoculation culture at an initial optical density of ~0.1 at 600 nm. The flasks were incubated at 37°C for 6 h without shaking and an RNase inhibitor (RNAlater, Ambion, TX, USA) added to prevent RNA degradation. The cells were harvested by centrifugation at 10,000 rpm for 7 min. Total RNA was isolated from the cells using the Qiagen RNeasy kit (Hilden, Germany). The harvested cells were lysed with glass beads and lysis buffer [1:100 ratio (*v*/*v*) of β-mercaptoethanol and RLT buffer], centrifuged, and the supernatant precipitated with chilled absolute ethanol. This mixture was passed through the gDNA eliminator spin column and washed twice with RW1 and RPE buffer before elution in nuclease-free water. The concentration and purity of RNA were assessed using the NanoVue Plus Nanodrop spectrophotometer (GE, Chicago, IL, USA). qRT-PCR was performed as previously described (Lee et al., 2021b) using the SYBR Green qPCR Master Mix (Applied Biosystems, Foster City, CA, USA) and the StepOne Real-Time PCR System (Applied Biosystems). The *RDN18* gene was used for housekeeping endogenous control. The gene-specific primers used in the study are provided in **Supplementary Table S2**. The fold change of gene expression was calculated as 2^(−dd*C*_t_).

### 2.7 Seed germination assay

The toxicity of NBP was assessed using seed germination of the winter cabbage *B. rapa*. The seeds were sorted for abnormalities, and the selected seeds were washed, soaked in distilled water for 8 h, and dried overnight. A total of 10 seeds per plate were placed on 0.7% soft agar of 0.86 g/L Murashige and Skoog (MS) plates and incubated at room temperature for 7 days. The root, stem, and total length of germinated seeds were calculated.

### 2.8 Toxicity assay using a *Caenorhabditis elegans* nematode model

The toxicity of NBP was assayed using a *C. elegans* model as previously described (Lee et al., 2021b). The *C. elegans* strain *fer-15(b26); fem-1(hc17)* was used. Synchronized adult worms were washed in M9 buffer (3 g/L KH_2_PO_4_, 6 g/L Na_2_HPO_4_, 5 g/L NaCl, and 1 mM MgSO_4_) before the start of the experiment. Approximately 30 worms in M9 buffer with or without NBP at concentrations of 2, 10, 20, 50, 100, 200, and 400 µg/ml were placed in 96-well plates and incubated for 10 days at 25°C without agitation. The percentage of live worms was determined by the responses of the worms to LED light exposure for 20–30 s using an iRiS Digital Cell Imaging System after incubation (Qian et al., 2024).

### 2.9 ADME profile of *N*-phthalimide derivatives

The toxicity, drug likeliness, and pharmacological properties of the *N-*phthalimide derivatives were profiled using online in silico tools including PreADMET (https://preadmet.qsarhub.com), GUSAR (http://www.way2drug.com/gusar), and Molinspiration (https://www.molinspiration.com), which were accessed on November 27, 2023. The properties of the derivatives were validated using server bioassay parameters or with a literature study. The complete ADME profile of the derivatives is provided in **Supplementary Table S3**.

### 2.10 Statistical analysis

All experiments were conducted with at least two independent cultures in triplicate. The results were indicated as the mean ± standard deviation. Student’s *t*-test was used to calculate statistical significance, and *p*-values ≤0.05 were considered significant. Graphs were drawn using SigmaPlot ver.14.0.

## 3 Results

### 3.1 Effect of phthalimide derivatives on biofilm formation, planktonic cell growth, and cell survival of *C. albicans*

**Figure 1:**
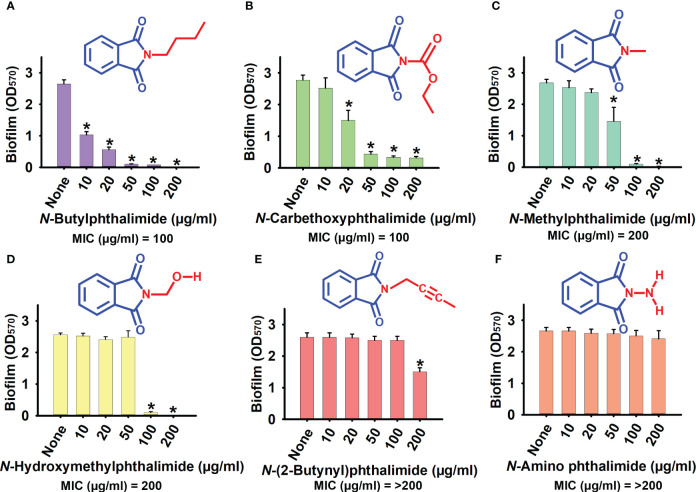
Effect of phthalimide derivatives on the biofilm formation of *Candida albicans* DAY185. **(A)** *N*-butylphthalimide. **(B)** *N*-carbethoxyphthalimide. **(C)** *N*-methylphthalimide. **(D)** *N*-hydroxymethylphthalimide. **(E)** *N*-(2-butynyl)phthalimide. **(F)** *N*-amino phthalimide. *C. albicans* cells were grown in potato dextrose broth (PDB) with and without treatment with *N*-butylphthalimide (NBP) at 37°C for 24 h. *p<0.05 vs untreated controls (None).

The antimicrobial and anti-biofilm activities of the six phthalimide derivatives were evaluated against *C. albicans* DAY185. Detailed information is presented in **Supplementary Table S1**. Of the derivatives examined, NBP was the most potent, which showed 61%, 78%, and 96% biofilm inhibition at concentrations of 10, 20, and 50 µg/ml, respectively (**Figure 1A**). The biofilm inhibition range of NBP of 60%–80% at the concentration range of 10–20 µg/ml is far superior to the 0%–45% range of the other derivatives at similar concentrations (**Figure 1**). The MIC of NBP was 100 µg/ml, which is similar to that of NCP, while NMP and *N*-(hydroxymethyl)phthalimide (NHMP) had MIC of 200 µg/ml. The MICs of N2BP and NAP were higher than the test range of 200 µg/ml (**Supplementary Table S1**).

**Figure 2:**
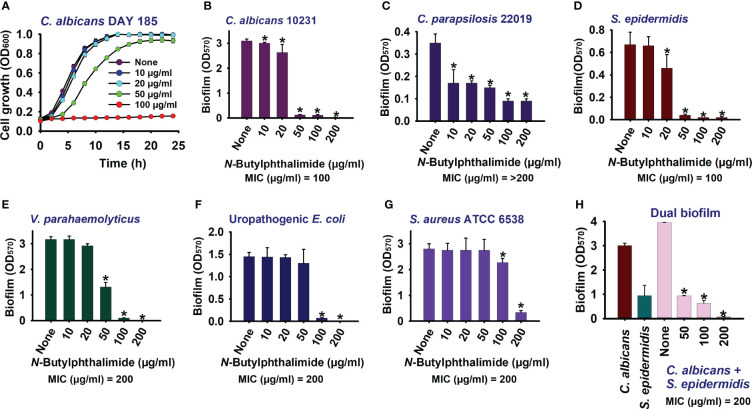
Impact of *N*-butylphthalimide (NBP) on the biofilm formation and planktonic cell growth of *Candida* species. By cultivating for 24 h at 37°C under static conditions in 96-well polystyrene plates, the planktonic cell growth of *Candida albicans* DAY185 **(A)** and the anti-biofilm activities of NBP against *C. albicans* 10231 **(B)**, *Candida parapsilosis* **(C)**, *Staphylococcus epidermidis* **(D)**, *Vibrio parahaemolyticus* **(E)**, uropathogenic *Escherichia coli* **(F)**, *Staphylococcus aureus* ATCC 6538 **(G)**, and polymicrobial biofilms **(H)** were investigated. *p<0.05 vs untreated controls (None).

NCP was also found to be a potent derivative, with biofilm inhibition of 45% and 84% at 20 and 50 µg/ml, respectively, and a MIC of 100 µg/ml (**Figure 1B**). However, NBP was chosen over NCP for its higher biofilm inhibition ability at lower test concentrations (**Supplementary Table S1**; **Figures 1A, B**). NBP inhibited biofilm formation by 97% at 100 µg/ml. At 10 and 20 µg/ml, NBP did not affect cell growth, with biofilm inhibition of 61% and 78%, respectively, prompting further tests against other virulence factors. NBP at 50 µg/ml inhibited cell growth only by a small fraction, whereas it completely inhibited growth at 100 µg/ml. At concentrations of 10, 20, and 50 µg/ml, NBP significantly inhibited biofilm formation, especially at 50 µg/ml with 96% biofilm inhibition without affecting cell growth, which minimizes the risk of resistance development by selective pressure (**Figures 1A**, **2A**).

NBP also dose-dependently inhibited biofilm formation in fluconazole-sensitive *C. albicans* 10231 and *C. parapsilosis* (**Figures 2B, C**). In fluconazole-sensitive *C. albicans* 10231, NBP was ineffective at the lower concentrations of 10 and 20 µg/ml, whereas it completely abolished biofilm formation at the higher concentrations of 100 and 200 µg/ml. Similarly, NBP was more effective at the higher concentrations of 100 and 200 µg/ml, with inhibition rates in the range of 25%–30% against the *C. parapsilosis* biofilm. NBP was also effective against bacteria such as UPEC, *S. aureus*, *S. epidermidis*, and *V. parahaemolyticus*, inhibiting their biofilms at the higher concentrations of 100 and 200 µg/ml (**Figures 2D–G**). NBP completely abolished biofilm formation in the aforementioned bacteria at 200 µg/ml, except for *S. aureus* in which only 87% of the biofilm was inhibited (**Figure 2G**). The MICs of NBP against all the tested panels of microorganisms are displayed in **Supplementary Table S4**. NBP had a similar MIC of 100 µg/ml against the *C. albicans* 10231 and *S. epidermidis* strains, but double the concentration against UPEC, *S. aureus*, and *V. parahaemolyticus* at 200 µg/ml. The MIC against *C. parapsilosis* was higher than 200 µg/ml.

Furthermore, polymicrobial biofilms involving fungi and bacteria are prevalent in various clinical scenarios. These mixed biofilms often exhibit increased resistance to antimicrobial agents. Hence, the anti-biofilm activity of NBP was determined against the polymicrobial biofilms of *S. epidermidis* and *C. albicans*. As expected, NBP dose-dependently inhibited dual biofilm formation and completely abolished biofilm formation at 200 µg/ml (**Figure 2H**).

The results of the cell survival assays showed that NBP is fungistatic or bacteriostatic rather than fungicidal or bactericidal (**Supplementary Table S1**). NBP at the MIC and 2× MIC concentrations did not kill cells in the various pathogens tested; therefore, it is fungistatic or bacteriostatic (**Supplementary Figure S1**). However, NBP, at these concentrations, completely abolished the biofilms, but did not affect cell survival, which showed similar growth at 0–24 h in the treated samples.

### 3.2 Hyphal inhibition of *C. albicans* on treatment with NBP

**Figure 3:**
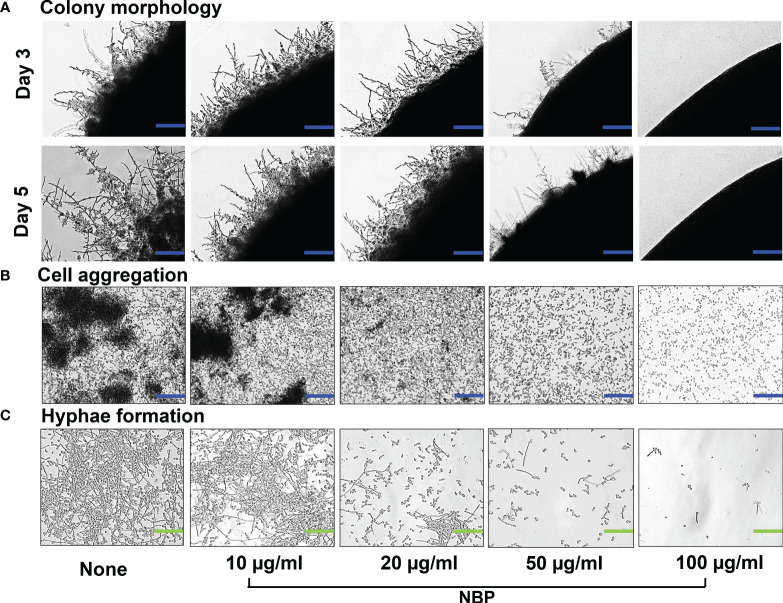
Morphogenesis of *Candida albicans* as impacted by *N*-butylphthalimide (NBP). **(A)** Colony morphology of *C. albicans* DAY185 on potato dextrose agar (PDA) solid medium at 37°C with and without NBP treatment on days 3 and 5 of growth. *Blue scale bars* indicate 100 µm. **(B, C)** Inhibition of cell aggregation **(B)** and hyphal formation **(C)** was assessed in potato dextrose broth (PDB) liquid medium after culture for 24 h. *Blue* and *green scale bars* represent 100 and 30 µm, respectively.

Since hyphal formation and cell aggregations are important prerequisites for biofilm formation, a hyphal protrusion assay was performed on solid agar and a yeast–hyphal transition along with cell aggregation was performed in a liquid medium. Hyphal protrusion was observed in non-treated colonies after 3 days of incubation, and NBP dose-dependently inhibited hyphal development as observed on days 3 and 5 (**Figure 3A**). At the treatment concentration of 100 µg/ml, NBP completely hindered hyphal development (**Figure 3A**). Cell aggregates entangled between hyphae after 24 h were observed in the non-treated control (**Figure 3B**). NBP treatment dose-dependently inhibited cell aggregation and filamentous growth (**Figures 3B, C**).

**Figure 4:**
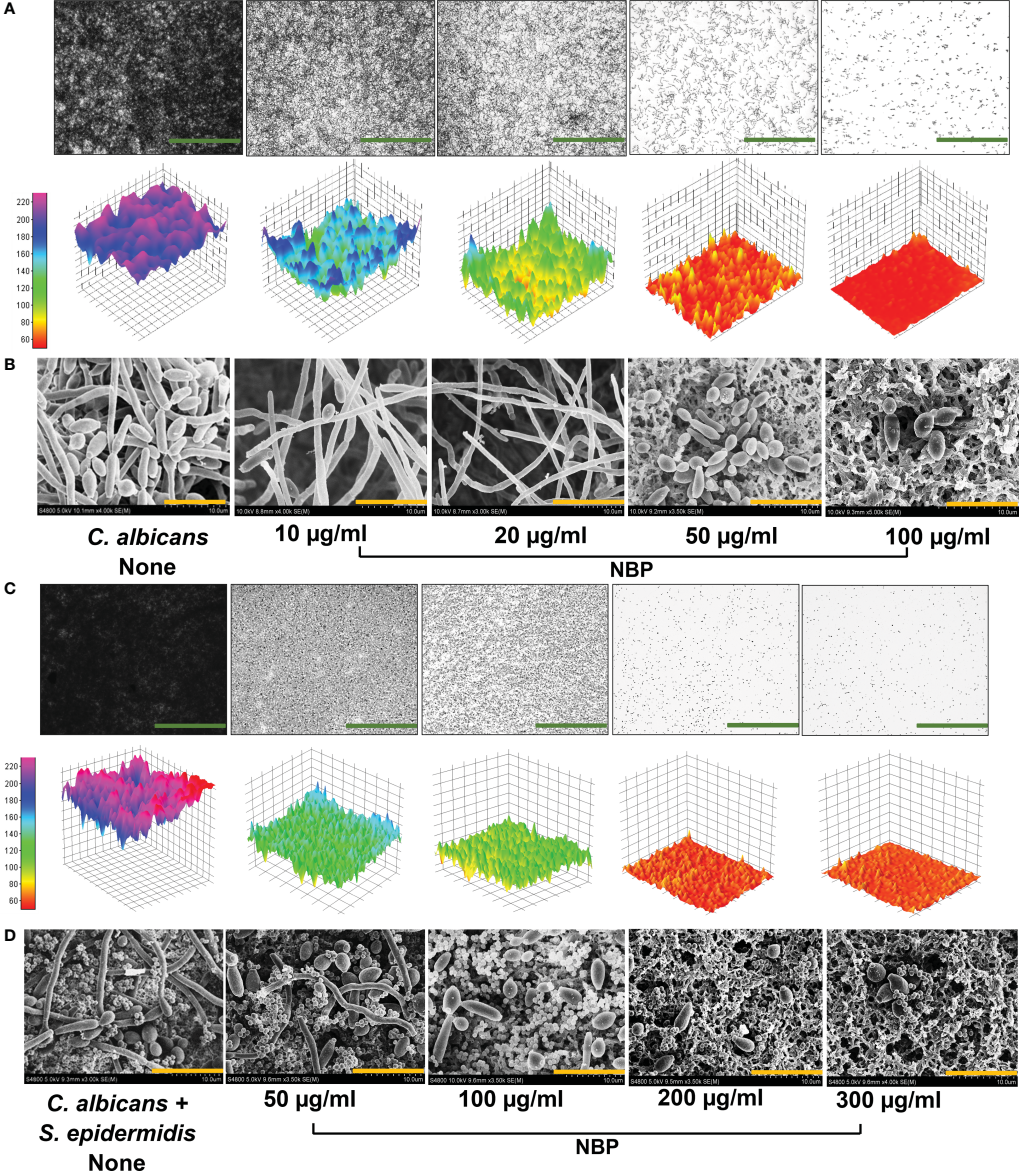
Observation of biofilm inhibition. **(A)** Biofilm inhibition of *Candida albicans* DAY185 displayed as 2D and 3D plots **(A)**. **(B)** Inhibition of hyphal growth in *C. albicans* DAY185 biofilms on nylon membranes visualized by SEM in the presence and absence of *N*-butylphthalimide (NBP). **(C, D)** Polymicrobial biofilm inhibition of *C. albicans* and *Staphylococcus epidermidis* displayed as 2D and 3D plots and visualized by SEM. *Green* and *orange scale bars* represent 100 and 10 µm, respectively. The *small round structures* are *S. epidermidis* cells, while the *bigger structures* are *C. albicans*.

The 2D and 3D visualization of the biofilm architecture of *C. albicans* also demonstrated significant biofilm inhibition by NBP at the concentrations of 10, 20, and 50 µg/ml, with complete abolishment at 100 µg/ml (**Figure 4A**). The non-treated control formed biofilms at the scale of 220, represented by the pink color. Treatment with NBP at concentrations of 10, 20, and 50 µg/ml inhibited biofilm formation to the 160 (cyan), 120 (green), and 80 (yellow) scales of the 3D representation, respectively (**Figure 4A**). At 100 µg/ml, NBP completely abolished biofilm formation to the 60 scale (red) (**Figure 4A**).

SEM analysis also demonstrated the hyphal inhibitory effect of NBP against *C. albicans* (**Figure 4B**). Non-treated controls demonstrated dense hyphal formation, and the combination of yeast cells with hyphae was observed (**Figure 4B**). NBP treatment caused a dose-dependent decrease in hyphal formation, with only a few scattered yeast cells on the nylon membrane at the higher treatment concentrations of 50 and 100 µg/ml (**Figure 4B**). Therefore, NBP dose-dependently inhibits hyphal and biofilm formation, as elucidated in the digital cell imaging and SEM analysis.

Visualization of the 2D and 3D architecture of the polymicrobial biofilms of two species *C. albicans* and *S. epidermidis* also demonstrated a dose-dependent biofilm inhibition with NBP treatment and the inhibition of biofilms to the 180 (blue), 100 (green), 70 (orange), and 60 (red) scales of 3D representation at concentrations of 50, 100, 200, and 300 µg/ml, respectively (**Figure 4C**). SEM analysis of the polymicrobial biofilms of *S. epidermidis* and *C. albicans* also demonstrated biofilm inhibition with NBP treatment. The abundance of both cells in the presence of NBP was decreased. The non-treated control showed significant hyphal formation by *C. albicans* interlaced with *S. epidermidis* cells, forming a dense biofilm matrix (**Figure 4D**). At concentrations of 50 and 100 µg/ml, NBP prevented hyphal formation in *C. albicans* with most yeast cells, while the *S. epidermidis* cells remained unaffected in the polymicrobial biofilm (**Figure 4D**). At concentrations of 200 and 300 µg/ml, NBP completely abolished polymicrobial biofilm formation (**Figure 4D**).

### 3.3 Gene expression in *C. albicans* post-NBP treatment

**Figure 5:**
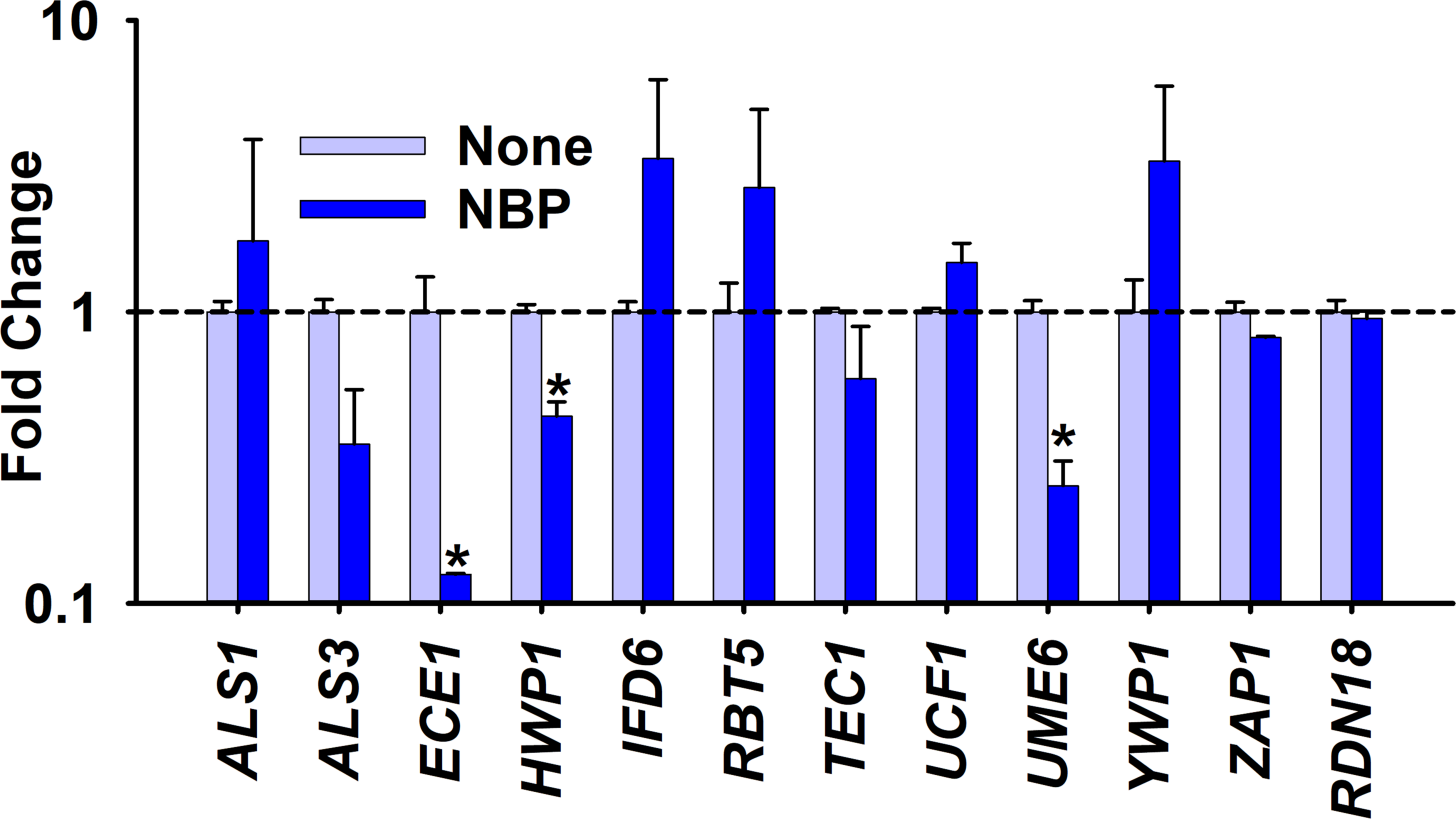
Gene expression changes by *N*-butylphthalimide (NBP) treatment. Relative transcriptional profiles of the biofilm- and hyphal-related genes of *Candida albicans* DAY185 with and without NBP treatment at 100 µg/ml for 6 h. *RDN18* was used as the housekeeping gene. *p < 0.05 *versus* non-treated controls.

To understand the molecular basis of the inhibition of biofilm formation and hyphal development, the transcriptional changes in 11 hyphal- and biofilm-related genes in *C. albicans* after treatment with NBP at 100 µg/ml were evaluated using qRT-PCR. NBP treatment downregulated six and upregulated five of the 11 examined genes. NBP treatment downregulated the expression of the agglutinin-like protein *ALS3* (2.8-fold), the hyphal-specific protein *ECE1* (8.0-fold), the hyphal cell wall protein *HWP1* (2.2-fold), and the filament-specific regulator *UME6* (4.0-fold). The housekeeping 18S RNA gene *RDN18* remained constant in both treated and untreated samples (**Figure 5**).

### 3.4 Seed germination and *C. elegans* toxicity assays

**Figure 6:**
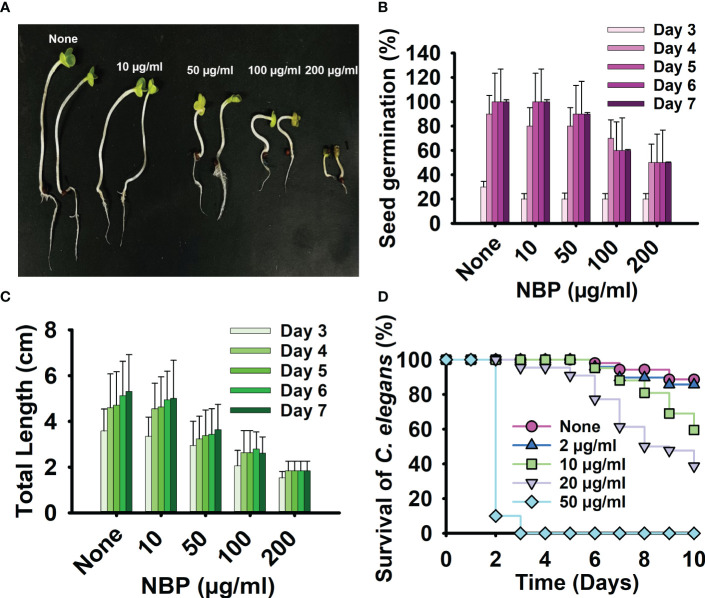
Toxicity of *N*-butylphthalimide (NBP) in a plant model and a nematode model. **(A)** The seed germination of *Brasicca rapa* was carried out at 25°C for 7 days using the Murashige and Skoog agar medium treated with or without NBP. **(B, C)** The seed germination rate **(B)** and the total length **(C)** were observed. **(D)** Survival of *C*. *elegans* observed with and without treatment of NBP at 25°C for 10 days.

The toxicity of NBP was evaluated using both seed germination of *B. rapa* and the *C. elegans* nematode model. NBP did not have any significant impact on seed germination at treatment concentrations of 10 and 50 µg/ml, with a marginal inhibitory effect on the length of the seedlings, the stems, and the roots (**Figures 6A–C**). However, NBP diminished seed germination and stunted the growth of the seedling at the higher testing concentrations of 100 and 200 µg/ml (**Figures 6A–C**). On visual inspection, the seedlings treated with higher concentrations of NBP, i.e., 100 and 200 µg/ml, also demonstrated a drier dark green appearance in comparison to the pale bright green appearance of the non*-*treated controls and the seedlings treated with lower NBP concentrations (**Figure 6A**).

The *C. elegans* nematode model was used to examine the toxicity of NBP for 10 days. NBP demonstrated nematicidal activity at all the concentrations tested, from 50 to 400 µg/ml, and killed all of the worms on day 1 of testing (**Figure 6D**). NBP was slightly toxic to worms at 2, 10, and 20 µg/ml and showed complete nematicidal activity (**Figure 6D**).

### 3.5 ADME profile of *N*-phthalimide derivatives

The comprehensive ADME profile of NBP along with NMP and NCP is documented in **Supplementary Table S3**. NBP presented a different ADME profile in comparison to the other phthalimide derivatives, NMP and NCP. NBP showed no Lipinski’s rule of five violations or lead-like violations and is suitable for lead-like rule. It also showed good plasma protein binding of 76%, which is significantly higher than that of the other derivatives, along with an excellent human intestinal absorption of 98%. In addition, NBP showed good lipophilicity with a miLog*P* value of 2.96 and a good topological polar surface area (TPSA) value of 39.08, showing decent membrane permeability. Furthermore, NBP was non-carcinogenic to mice and was non-toxic to medaka and minnow fish. It did not show severe toxicity to rat and belong to class 4 on the LD_50_ classification of the intraperitoneal (IP), intravenous (IV), oral, and subcutaneous (SC) routes of administration. NBP is also a negative protein, enzyme, and kinase inhibitor and is an ion channel modulator with a low risk of *hERG* (human ether-a-go-go related gene) inhibition (**Supplementary Table S3**).

## 4 Discussion

Phthalimide is a versatile pharmacological scaffold with anticancer, anti-inflammatory, antiparasitic, and, importantly, antimicrobial activities (Fernandes et al., 2023). Phthalimide and its derivatives also have various applications in the cosmetic and biomedicine industries (Durairaju et al., 2021). *N*-substituted phthalimides have exhibited antifungal activity against Aspergillus niger and Aspergillus flavus (Neelottama Kushwaha, 2016), and novel heterocycle-substituted phthalimides are potent against *C. albicans* (Jelali et al., 2022). The current study is the first to present the antifungal, anti-biofilm, and anti-hyphal activities of NBP against *C. albicans*.

Among the examined *N*-substituted phthalimides, NBP was shown as the most potent derivative (**Supplementary Table S1**). NBP dose-dependently inhibited the cell growth, biofilm formation, hyphal development, and cell aggregation and altered the colony morphology of *C. albicans* (**Figures 1**–**4**). NBP was also effective against other Candida species such as the fluconazole-sensitive *C. albicans* and *C. parapsilosis* (**Figures 2B, C**), and it has the potential to be a singular therapeutic for candidiasis originating from diverse pathogens. Furthermore, NBP inhibited biofilm formation in Gram-positive *S. aureus* and *S. epidermidis* and Gram-negative UPEC and *V. parahaemolyticus* without affecting cell survival (**Figures 2D–G**; **Supplementary Figure S1**). It was also effective in inhibiting the polymicrobial biofilms of S. epidermidis and *C. albicans* (**Figures 2H**, **4C, D**). It is interesting to note that NBP, at a concentration of 50 µg/ml, was effective in inhibiting the polymicrobial biofilms of *S. epidermidis* and *C. albicans*, but was ineffective against the mono-species biofilms of UPEC and S. aureus. This could be due to the increased lipid content in the fungal cell wall of *C. albicans* allowing better membrane permeability compared to the bacterial cell walls (Nayab et al., 2015). NBP can be an effective broad-spectrum therapeutic option with confirmed activity against Gram-negative and Gram-positive bacteria and fungi with a lower risk of resistance development owing to its microbe-static mode of action. This broad-spectrum microbe-static mode of action can plausibly be a result of the disruption of a common microbe cell replication pathway. There are several studies in which derivatives with phthalimide cores have demonstrated such cell replication inhibition via various mechanisms including DNA binding (Nayab et al., 2017; Arif et al., 2019), transcriptase or polymerase inhibition (Cingolani et al., 1976; Ungwitayatorn et al., 2008; Penta et al., 2013; El Hassab et al., 2024), DNA gyrase inhibition (Kamal et al., 2006; Zahran et al., 2018; Othman et al., 2019), and folate inhibition (Catalano et al., 2016). A more comprehensive study is required to ascertain the exact mechanism of the microbe-static activity of NBP against broad-spectrum microbes.

Furthermore, NBP repressed the gene expression of several biofilm- and hyphal-related genes (**Figure 5**). NBP significantly downregulated the hyphal-specific adhesin HWP1, which promotes adherence for biofilm formation and hyphal mass, along with UME6, which aids in filamentous growth (Nobile et al., 2006; Banerjee et al., 2008). NBP most significantly downregulated the ECE1 gene, which is responsible for hyphae and the production of the fungal toxin peptide candidalysin (Liang et al., 2024).

The potency of NBP can be partially attributed to the *N*-butyl substitution, which made the scaffold more hydrophobic as expected for alkyl substitutions, increasing interactions with biomolecules (Sahoo et al., 2019). This was also observed in another study wherein the N-pentyl substitution of phthalimide displayed the best antifungal activity against *C. albicans* (Jelali et al., 2022). Our results are partially supported by another study on the hypolipidemic biological activities of the chemically modified N-substituted phthalimides in which alkyl and alkanoic acid substitutions of up to five carbon atoms significantly increased bioactivity, whereas the substitution of other substituents such as the hydroxy, amino, and carbethoxy groups decreased activity (Chapman et al., 1983). In addition, *N*-substituted phthalimides demonstrated antifungal activity against Botrytis cinerea and Alternaria solani, in which the structure–activity relationship revealed that appropriate alkyl chain substitutions increased the antifungal efficiency of phthalimides (Pan et al., 2016). The chain length of antifungal compounds is also relevant against *C. albicans*, wherein medium-chain fatty acids mimicking farnesol were more effective than short-chain and long-chain fatty acids (Lee et al., 2021a). This might explain better the antifungal activity of NBP over other derivatives such as NCP and NMP. The better plasma protein binding and membrane permeability of NBP in comparison to NMP and NCP could have also contributed to its better antifungal activity (**Supplementary Table S3**).

**Figure 7:**
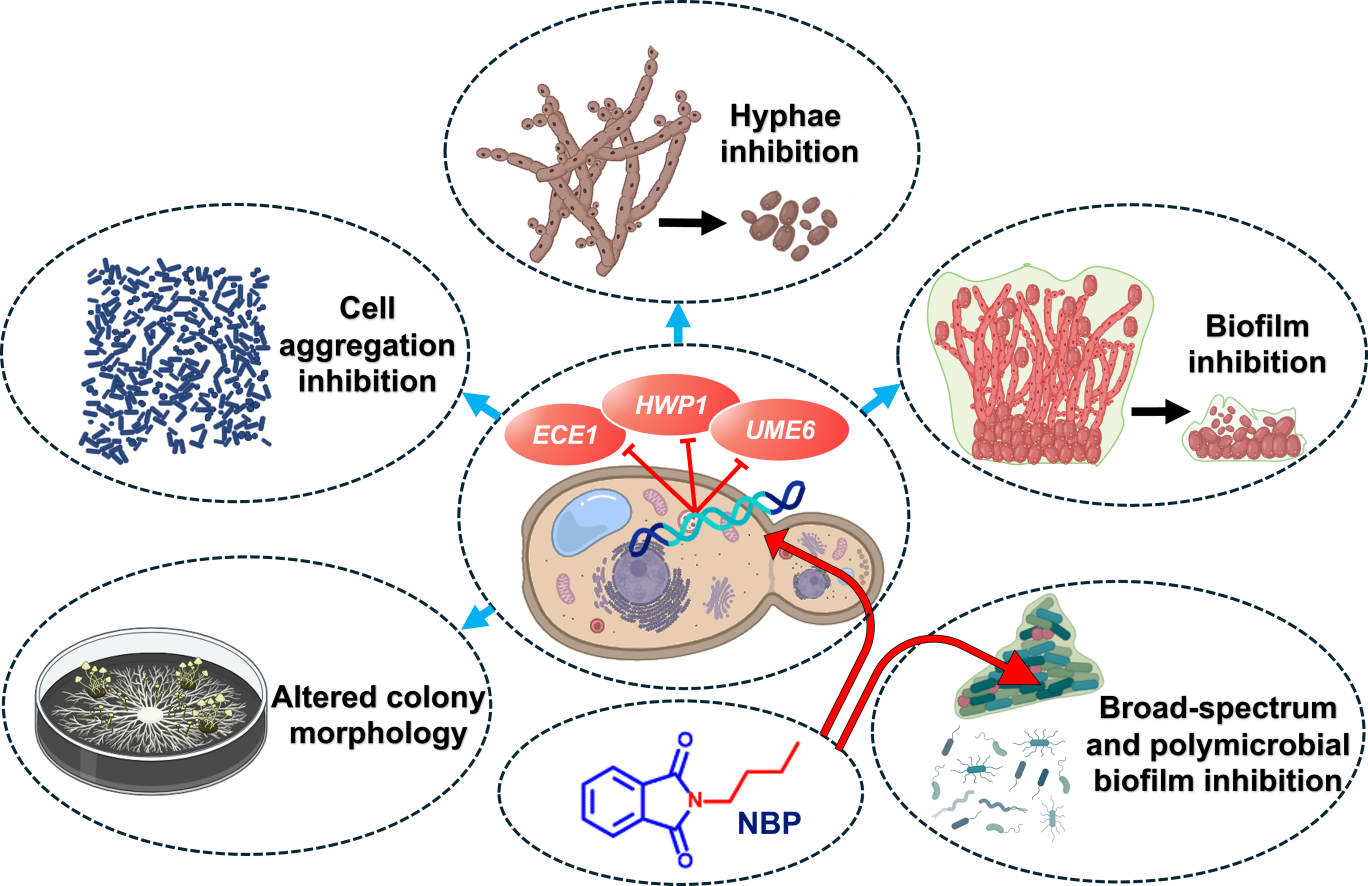
Graphical presentation of the anti-biofilm and anti-virulence effects of *N*-butylphthalimide (NBP) against *Candida albicans*.

While NBP exhibited toxicity in the nematode assay (**Figure 6D**), phthalimide derivatives hold significant promise in the cosmetic and biomedical fields (Durairaju et al., 2021). Their hydrophobic and neutral qualities make them particularly advantageous for cosmetic formulations, providing distinct benefits such as antioxidant, anti-inflammatory, and antimicrobial properties (Durairaju et al., 2021). The current study reports for the first time the strong antifungal and anti-biofilm activities of NBP (**Figure 7**).

## 5 Conclusion

This is the first study to report the anti-biofilm and anti-hyphal properties of *N*-substituted phthalimide derivatives against *Candida* species. Of the examined derivatives, NBP was the most potent and was fungistatic against *C. albicans*. These derivatives also inhibited the biofilm formation of other pathogenic bacteria such as UPEC, *S. epidermidis*, *S. aureus*, and *V. parahaemolyticus*, along with the polymicrobial biofilms of *S. epidermidis* and *C. albicans*. They also significantly inhibited hyphal formation and cell aggregation and altered the colony morphology in *C. albicans*. NBP downregulated the important *C. albicans* biofilm- and hyphal-related genes *ECE1*, *HWP1*, and *UME6.* Therefore, NBP could be a promising candidate for therapy against candidiasis, which also has the potential to serve as a broad-spectrum anti-biofilm agent against both Gram-positive and Gram-negative bacteria.

## Data availability statement

The original contributions presented in the study are included in the article/**Supplementary Material**. Further inquiries can be directed to the corresponding author.

## Author contributions

SS: Conceptualization, Data curation, Formal analysis, Investigation, Methodology, Visualization, Writing – original draft. J-HL: Data curation, Formal analysis, Funding acquisition, Methodology, Project administration, Resources, Supervision, Writing – review & editing. Y-GK: Funding acquisition, Methodology, Resources, Writing – review & editing. JL: Data curation, Formal analysis, Project administration, Resources, Supervision, Validation, Visualization, Writing – review & editing.
