# Supplementary material for: Antifungal, anti-biofilm, and anti-hyphal properties of N-substituted phthalimide derivatives against Candida species
Source: Front Cell Infect Microbiol. 2024 Jun 5;14:1414618. doi: 10.3389/fcimb.2024.1414618 (PMC11188339; doi:10.3389/fcimb.2024.1414618)
Supplement: Supplementary file 1 [file DataSheet_1.docx]

**Supplementary Material**

**Antifungal, antibiofilm and anti-hyphal properties of *N-*substituted phthalimide derivatives against *Candida* species**

Shamshe Shaik, Jin*-*Hyung Lee, Yong-Guy Kim and Jintae Lee*

School of Chemical Engineering, Yeungnam University, Gyeongsan, Republic of Korea

* Corresponding author: [jtlee@ynu.ac.kr](mailto:jtlee@ynu.ac.kr), Tel.: +82-53-810-2533, Fax: +82-53-810-4631

Running title: Antibiofilm activity of phthalimides**Table S1.** Inhibition of biofilm formation and planktonic cell growth by six phthalimide derivatives against *C. albicans* at concentrations 10, 20, 50 and 100 µg/ml. MICs (minimum inhibitory concentrations) were defined as the lowest concentrations that prevented fungal growth. The % biofilm and cell growth inhibition is defined as the percentage reduction of biofilm formation and cell growth after treatment compared to the control”.

| Chemical names | Structures | MIC µg/ml | Biofilm inhibition (%) | | | | Cell growth (%) | | | |
| --- | --- | --- | --- | --- | --- | --- | --- | --- | --- | --- |
|  |  |  | **10 µg/ml** | **20 µg/ml** | **50 µg/ml** | **100 µg/ml** | **10 µg/ml** | **20 µg/ml** | **50 µg/ml** | **100 µg/ml** |
| *N*-Butylphthalimide |  | 100 | 61.1 | 78.9 | 96.2 | 97 | 2.9 | 4.8 | 11.5 | 83.6 |
| *N*-Carbethoxyphthalimide |  | 100 | 9.0 | 45.8 | 84.1 | 87.7 | 0.0 | 0.0 | 3.7 | 93.6 |
| *N*-Methylphthalimide |  | 200 | 5.6 | 11.6 | 45.5 | 96.3 | 0.0 | 0.0 | 1.9 | 77.8 |
| *N*-Hydroxymethylphthalimide |  | 200 | 1.6 | 5.9 | 2.7 | 96.1 | 0.9 | 1.9 | 2.9 | 71.2 |
| *N*-(2-Butynyl)phthalimide |  | >200 | 0.0 | 0.8 | 3.9 | 3.8 | 0.0 | 0.0 | 0.0 | 0.9 |
| *N*-Amino phthalimide |  | >200 | 0.0 | 2.6 | 3.0 | 5.6 | 1.9 | 1.9 | 1.9 | 1.9 |

**Table S2.** Primers used in the qRT-PCR study.

| **Gene** | **Primer** |
| --- | --- |
| *ALS1* | Forward 5'-AGC TGT TGC CAG TGC TTC-3' |
|  | Reverse 5'-AAT GTG TTG GTT GAA GGT GAG-3' |
| *ALS3* | Forward 5'-CAA CAT CAA CCA ACC AAT CTC-3' |
|  | Reverse 5'-TGA ATA ACA GAA CCA GAT CCG-3' |
| *ECE1* | Forward 5'-CCA GAA ATT GTT GCT CGT GTT GCC A-3' |
|  | Reverse 5'-TCC AGG ACG CCA TCA AAA ACG TTA G-3' |
| *HWP1-1* | Forward 5'-TGG TGC TAT TAC TAT TCC GG-3' |
|  | Reverse 5'-CAA TAA TAG CAG CAC CGA AG-3' |
| *RBT5* | Forward 5'-CTG CTG AAA GTT CTG CAC CA-3' |
|  | Reverse 5'-GCT TCA ACG GAA ACA GAA GC-3' |
| *TEC1* | Forward 5'-AGG TTC CCT GGT TTA AGT G-3' |
|  | Reverse 5'-ACT GGT ATG TGT GGG TGA T-3' |
| *UCF1* | Forward 5'-ATG GCG GGA AAG AAA AAG TC-3' |
|  | Reverse 5'-CCC AAG TTT CAT CAC GAA CA-3' |
| *UME6* | Forward 5'-AGC ACC AAA TTC GCC TTA TG-3' |
|  | Reverse 5'-AGG TTG AGC TTG CTG CAG TT-3' |
| *YWP1* | Forward 5'-GTT CCA TTT TTC CAA GTT CAT TTA G-3' |
|  | 5'-TCA AGA GTA GAA CCT TCA AGA GCA G-3' |
| *ZAP1* | Forward 5'-CGA CTA CAA ACC ACC AGC TTC ATC-3' |
|  | Reverse 5'-CCC CTG TTG CTC ATG TTT TGT T-3' |
| *RDN18* | Forward 5'-AGA AAC GGC TAC CAC ATC CCA-3' |
|  | Reverse 5'-CGA ATG GGC CCT GTA TCG T -3' |

**Table S3.** ADME (absorption, distribution, metabolism, and excretion) profile of *N*-butyl phthalimide (NBP), *N*-carbethoxy phthalimide (NCP), *N*-methyl phthalimide (NMP), *N*-hydroxymethylphthalimide (NHP), *N*-(2-butynyl) phthalimide (N2BP), *N*-amino phthalimide (NAP) .

| **Property** | **NBP** | **NCP** | **NMP** | **NHP** | **N2BP** | **NAP** |
| --- | --- | --- | --- | --- | --- | --- |
| Lead like rule | Suitable if its binding affinity is greater than 0.1 microM | Suitable if its binding affinity is greater than 0.1 microM | Violated | Violated | Suitable if its binding affinity is greater than 0.1 microM | Violated |
| Lead like violations | 0 | 0 | 1 | 1 | 0 | 1 |
| Lipinski’s rule of five violations | 0 | 0 | 0 | 0 | 0 | 0 |
| Plasma protein binding | 76.82 | 63.54 | 32.33 | 24.64 | 69.3 | 17.72 |
| Blood brain barrier permeability | 2.36 | 0.89 | 1.151 | 0.42 | 2.23 | 0.32 |
| Skin absorption | -2.84 | -3.70 | -2.85 | -3.89 | -3.18 | -3.6 |
| Human intestinal absorption | 98.47 | 96.96 | 98.55 | 94.71 | 98.40 | 95.41 |
| Caco2 | 22.56 | 21.11 | 21.12 | 20.96 | 22.82 | 20.35 |
|  |  |  |  |  |  |  |
| invitro HERG inhibition | Low risk | Low risk | Low risk | Low risk | Low risk | Low risk |
| miLogP | 2.96 | 1.75 | 1.52 | 0.88 | 2.50 | 0.91 |
| mol volume | 190.9 | 185.2 | 140.5 | 148.77 | 179.46 | 135.24 |
| tpsa | 39.08 | 65.38 | 39.08 | 59.30 | 39.08 | 65.10 |
| GPCR ligands | -0.45 | -0.44 | -0.99 | -0.84 | -0.24 | -1.21 |
| Ion channel modulator | -0.38 | -0.03 | -0.65 | -0.51 | -0.41 | -0.74 |
| Kinase inhibitor | -0.63 | -0.93 | -0.91 | -0.86 | -0.70 | -0.88 |
| Nuclear receptor ligand | -0.81 | -0.35 | -1.15 | -1.04 | -0.66 | -1.72 |
| Protein inhibitor | -0.66 | -0.66 | -1.10 | -1.10 | -0.15 | -1.39 |
| Enzyme inhibitor | -0.14 | -0.10 | -0.59 | -0.29 | -0.11 | -0.51 |
| Mouse carcinogenicity | negative | Positive | negative | negative | negative | negative |
| Acute fish toxicity (medaka) | 0.13 | 0.15 | 1.01 | 1.76 | 0.1 | 2.07 |
| Acute fish toxicity (minnow) | 0.07 | 0.09 | 0.52 | 1.04 | 0.05 | 0.84 |
| Rat IP LD_50_ classification | Nontoxic out of AD | Class 5 in AD | Class 5 in AD | Class 5 in AD | Class 4 in AD | Class 4 in AD |
| Rat IV LD_50_ classification | Class 4 in AD | Class 4 in AD | Class 4 in AD | Class 4 in AD | Class 3 in AD | Class 4 in AD |
| Rat Oral LD_50_ classification | Class 4 in AD | Class 4 in AD | Class 4 in AD | Class 4 in AD | Class 4 in AD | Class 4 in AD |
| Rat SC LD_50_ classification | Class 4 in AD | Class 4 in AD | Class 5 in AD | Class 4 in AD | Class 4 in AD | Class 4 in AD |

**Table S4.** MICs and % biofilm inhibition of *N*-butylphthalimide against pathogenic microbes used in the study.

| **Strain names** | **MIC µg/ml** | **Biofilm inhibition (%)** | | |
| --- | --- | --- | --- | --- |
|  |  | **50 µg/ml** | **100 µg/ml** | **200 µg/ml** |
| ***C*. *albicans* DAY185** | 100 | 96.2 | 97 | 99.6 |
| ***C*. *albicans* 10231** | 100 | 96.1 | 96.4 | 98.3 |
| ***C*. *parapsilosis* 22019** | >200 | 57.4 | 74.2 | 74.2 |
| ***S*. *epidermidis*  ATCC 14990** | 100 | 94.0 | 97.0 | 97.0 |
| ***V*. *parahaemolyticus* ATCC 17802** | 200 | 58.6 | 96.8 | 99.6 |
| **Uropathogenic *E*. *coli* CFT073** | 200 | 10.3 | 95.1 | 99.3 |
| ***S*. *aureus* ATCC 6538** | 200 | 1.7 | 18.9 | 87.8 |

**Fig. S1.** Cell survival assay of *C. albicans*, *S. aureus*, UPEC and *V. parahaemolyticus*

**
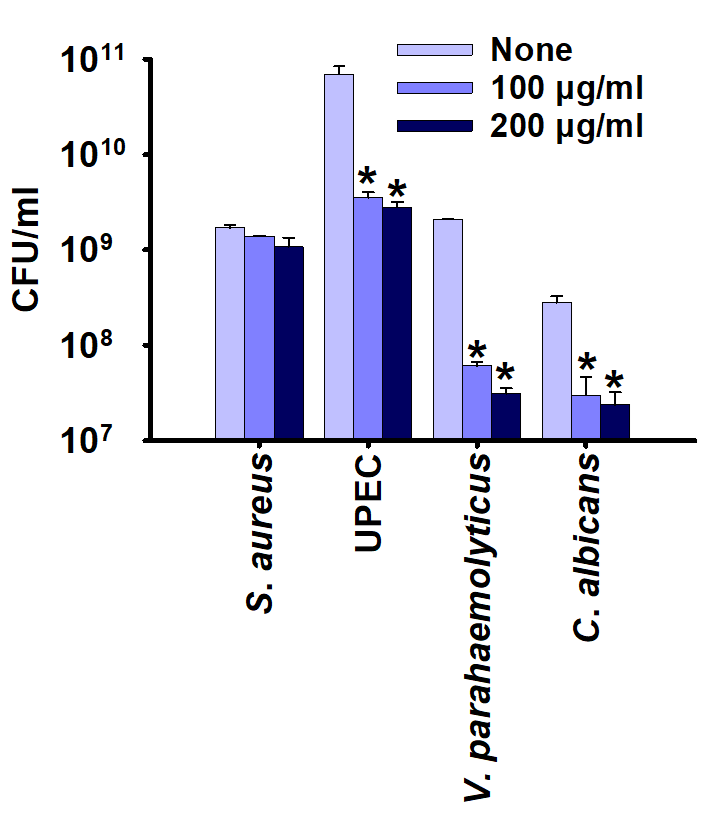
**
